# Determinants of Initial Utilization of Community Healthcare Services among Patients with Major Non-Communicable Chronic Diseases in South China

**DOI:** 10.1371/journal.pone.0116051

**Published:** 2014-12-29

**Authors:** Huajie Yang, Xiang Huang, Zhiheng Zhou, Harry H. X. Wang, Xinyue Tong, Zhihong Wang, Jiaji Wang, Zuxun Lu

**Affiliations:** 1 School of Public Health, Tongji Medical College of Huazhong University of Science and Technology, Wuhan, 430030, China; 2 School of Public Health, Guangzhou Medical University, Guangzhou, 510182, China; 3 School of Public Health and Primary Care, Faculty of Medicine, The Chinese University of Hong Kong, New Territories, Hong Kong; 4 General Practice and Primary Care, Institute of Health and Wellbeing, University of Glasgow, Glasgow, G12 9LX, United Kingdom; 5 The Affiliated Liyuan Hospital of Tongji Medical College, Huazhong University of Science and Technology, Wuhan, 430077, China; Alberta Provincial Laboratory for Public Health/ University of Alberta, Canada

## Abstract

**Background:**

Although expected to act as gate-keeping primary care providers, as community health service (CHS) facilities are severely under-utilized; Chinese people in both rural and urban areas used predominantly higher-tier facilities for primary care purpose, with significant financial and outcome consequences. This study intends to explore the determinants of initial utilization of CHS among patients with major non-communicable chronic diseases (NCDs) in order to understand the care-seeking behavior among urban and rural residents in South China.

**Methods:**

A multi-stage cluster random sampling methodology was adopted to create a sample of 19,466 adults with NCDs from 7,970 urban households and 32,035 adults with NCDs from 3,860 rural households in Guangdong, China. Interviews and physical examinations were conducted in 2010 to collect data on patient characteristics, medical conditions, and awareness and utilization of healthcare. Descriptive analysis and logistic regression analysis were performed to study utilization patterns and the factors associated with the patterns.

**Results:**

Prevalence of major NCDs in urban areas was significantly higher than that in rural areas (12.55% vs. 8.70%; *p*<0.001). Second-tier district hospitals were most preferred for initial consultation (46.05% in rural areas vs. 45.32% in urban areas; *p*<0.001), followed by tertiary general or specialized hospitals (28.39% in rural areas vs. 33.89% in urban areas; *p*<0.001). The proportion of patients who had initial use of CHS was relatively low (25.56% in rural areas vs. 20.79% in urban areas; *p*<0.001). Awareness of self-care and the presence of medical insurance were leading factors associated with first contact of CHS facilities in both urban and rural areas.

**Conclusion:**

The study suggests that CHS facilities are not often used as the first contact for patients in both rural and urban areas in south China. Much effect must be made to enhance the gatekeeper system and improve medical insurance coverage in future healthcare reforms.

## Introduction

There are two recognized models that dictate a patient’s access to health care: the gatekeeper system and the direct-access system [1]. In a gatekeeper system, medical insurance covers initial consultation with general practitioners (GPs). In contrast, the direct-access system grants patients access to all medical care, including both specialists and GPs. The gatekeeper system has often been demonstrated as advantageous in moderating use of resources, controlling healthcare expenditure, improving disease diagnosis and treatment [2], and reducing disparities in healthcare access [3]. There is considerable variation in the range of services offered by GPs [4], and the differences are associated with features of the healthcare system, such as the mode of patient access to secondary care (direct or on referral by GPs in a gatekeeper role), remuneration method (fees for items of service, capitation fees or salary) and healthcare supply characteristics (density of GPs and of specialists) [5]–[7]. Many studies have shown that when patients are provided with choices and GPs act as gatekeepers to secondary care, the costs were lower, patient satisfaction higher, health status better, and reliance on medication is less [8], [9] [10] [11] [12]. Not surprising, the World Health Organization (WHO) has long emphasized the importance of the gatekeeper system as an organizational model for structuring healthcare system.

Unfortunately, owing to many historic reasons, China has been utilizing the second model, the direct-access model, forgoing the potential benefits of a gatekeeper system. Although Chinese government has repeatedly stressed the importance of community health services (CHS) facilities in primary care, no restriction is made in patient’s freedom to select all medical facilities and doctors in terms of policies and health insurance coverage. Consequently, too often patients go directly to higher-tier, more sophisticated or specialized, more expensive hospitals instead of community health centers or stations for primary care. This care-seeking pattern is particularly of concern among people of advanced age or with chronic conditions [13] [14] [15], [16] [17]. Recognizing this problem [18], the government started a pilot project to test a system of initial treatment in CHS centers in Beijing, Shenzhen, Nanjing, Nanning, and other cities in 2006 [19]. Furthermore, the Chinese government has undertaken a new round of healthcare reforms, focusing on strengthening the primary healthcare service system, encouraging the use of CHS centers for first contact of care, and improving cooperation and care transition between CHS facilities and higher-tier, secondary hospitals with two-way referrals (i.e., CHS facilities transfer patients to hospitals for more advanced care and hospitals transfer patients back to CHS centers for rehabilitation).

However, there is currently no well-established and unified initial treatment system in the community and relevant supporting medical insurance system in most regions, and patient can choose among many medical institutions for initial treatment. Moreover, the long-standing divide between urban and rural areas results in an unequal distribution of healthcare resources. Thus, the establishment of an initial treatment system is an effective mechanism for narrowing the gap between urban and rural areas, consequently promoting healthcare equality. However, study on healthcare-seeking behaviors have been confined to a specific area or patients with certain types of diseases and there is definite absence of comparative studies investigating the difference between rural and urban areas in China [20].

We hypothesize that there are significant differences in determining factors of healthcare-seeking behaviors between rural and urban areas. This study focused on patients with five major non-communicable chronic diseases (NCDs), analyzed their healthcare-seeking patterns and determining factors in both rural and urban areas. Our findings will help policy makers to establish and improve an initial treatment system, eliminate disparities between regions, and promote healthcare equality between urban and rural areas.

## Methods

### Study Design and Subjects

As one of the most economically developed areas in China, Guangdong province has a relatively mature medical infrastructure and public health system, and, in particular, a well-developed CHS network. Note that, in China, hospitals can be classified into three different classes according to their major functions. The first-tier hospitals generally include township hospitals and community health centers. Since 2009, the health authorities have gradually transformed township hospitals into community health centers that provide basic medical care, prevention, rehabilitation, health education, and family planning services to residents within a certain area. The second-tier hospitals include those that provide comprehensive medical and healthcare services and are able to undertake teaching and research tasks within a district. Generally, hospitals in county, district, and cities are at or above secondary level. The tertiary hospitals include those that provide healthcare service trans-regionally and nationwide, equipped with medical care, teaching, and research capacities. This study included all these hospitals, with a focus on community health centers.

This study used a multi-stage cluster random sampling method to select 5%–10% of the total resident population of Guangdong province in south China. In the first stage, nine cities were selected, including two cities in the west, two in the north, two in the east, and three in the central region. In the second stage, one community administrative service center (CASC) with the CHS facility in its jurisdiction was chosen randomly according to the registered code in each city. In the third stage, five communities were selected within the jurisdiction of each CASC. With this sampling method, nine CASCs were selected randomly with a total of 45 communities. Inclusion criteria stipulated that participants must be residents who have lived in these communities for at least six months, including registered and nonregistered residents. The number of participants had to be no less than 10% of the population served by the selected CASC. If the number did not reach the pre-defined level, a completely random sampling method was adopted to supplement the insufficient sample size. Thereafter, researchers interviewed participants at their homes.

Baseline data were collected using questionnaires and physical examinations from March to September 2010. The design combined both qualitative (observation and interview) and quantitative (questionnaire survey) measures. The questionnaire was a slightly modified version of the questionnaire that was well validated and broadly used previously [21], which included residents’ basic demographic information, basic knowledge of health, presence of chronic diseases, and the awareness and utilization of CHS. Each subject was interviewed with their families by well-trained interviewers and community medical staff. The community physicians and nurses were responsible for conducting physical examinations. Healthcare outcome was defined as self-reported chronic disease that was either diagnosed previously at any hospital or identified on-site during the study for the first time.

The investigators followed pre-defined protocol to conduct interview. First, they introduced the purpose of the survey and explained the contents of the questionnaire. All participants read a statement that explained the purpose of the survey and provided written informed consent before participating in the study. Then, anonymous questionnaires were used for on-site interviews conducted by trained physicians and nurses for patients.

### Measurement Tools

This study focused on the five major NCDs with high community prevalence and a substantial, negative impact on public health, namely, hypertension, diabetes, stroke, heart disease, and chronic obstructive pulmonary disease (COPD). Participants with chronic diseases were defined as those who were diagnosed with at least one of the listed NCDs in a hospital, or those who were diagnosed with an NCD during the survey. For this study, which covered a wide range of districts and residents, patients with NCDs were primarily identified using self-report in a hospital, particularly for diabetes, COPD, stroke, and heart disease. The physical examinations mostly diagnosed hypertension, as it is easily diagnosed in this way. With these two methods, we sought to find and collect as many patients with NCDs as possible. These NCDs were diagnosed according to the disease classification standard of outpatient service in the government annual report established by the Chinese Ministry of Health. According to the WHO-ISH (World Health Organization International Society of Hypertension) guidelines for Hypertension Management 1999, essential hypertension (EHT) was defined as a systolic blood pressure (SBP) ≥140 mmHg and/or a diastolic blood pressure (DBP) ≥90 mmHg. Patients with diabetes, COPD, stroke, and heart disease were determined using diagnostic criteria adopted by relevant hospitals.

### Statistical methods

All questionnaires were checked for completeness, and the EpiData software (version 3.1) was used for data entry. Double entry was conducted to ensure data accuracy. The SAS system (8.2, SAS Institute Inc., Cary, NC, USA) was used for data cleaning and analysis. The data were presented as rates, and differences between groups examined using the chi-square test. All alpha levels were set to 0.05. An unconditional logistic regression model was used to analyze the choice of medical facilities by patients with NCDs in urban and rural areas. Variable selection for multiple regression analyses was conducted using a stepwise entry process, and visiting a CHS facility for treatment was used as the dependent variable (1 = yes, 0 = no). A univariate logistic regression analysis was conducted, considering nine related factors including age, gender, marital status, education level, occupation, exercise, healthcare, and community health knowledge, utilization rate of CHS, and time to visit CHS. Factors demonstrated to be statistically significant in the univariate analysis were entered into a multivariate unconditional logistic regression as independent variables, and the backward LR method was used to build the regression model, with 0.05 as the inclusion standard and 0.10 as the exclusion standard. Age, gender, marital status and others were classified as demographic factors; education, physical exercise, healthcare knowledge, and community health care report as awareness factors; occupation, income level, medical insurance, and healthcare expenditure as socioeconomic factors.

## Results

### Basic demographic information

The number of selected subjects was 19,466 for urban, and 32,035 for rural regions. The study demonstrated that people aged 65 years or above in both urban and rural communities constituted more than 10% of the total population. The proportion of residents with lower income was larger in rural than in urban areas. Annual expenditure for medical diagnosis and treatment was 3,666.79 RMB for urban residents, whereas most residents living in rural area spent less than 1,000 RMB per year. The study showed that in rural areas, 92.2% of the subjects knew that there was a CHS center or station close to their residence. They could reach the CHS facilities within an average time of 9.53 minutes. In urban areas, however, only 75.4% of the subjects knew about the CHS center or station, and the average time to reach the CHS facilities was 13.04 minutes. There was a significant difference in gender, age, marital status, education, occupation, family per capita monthly income, and medical insurance between urban and rural residents (*P<*0.001). [chi-squared test] (Table 1).

**Table 1:** Demographic information of study samples from rural and urban areas (N = number).

Items	Groups	Urban(N = 19466)		Rural(N = 32035)		?^2^	*P* value
		n	%	N	%		
Gender	Male	9445	48.52	15881	49.57	5.38	<0.001
	Female	10021	51.48	16154	50.43		
Age(year)	18∼24	2660	13.66	5321	16.61	351.94	<0.001
	25∼34	4571	23.48	5873	18.33		
	35∼44	4210	21.63	6343	19.80		
	45∼54	2874	14.77	5821	18.17		
	55∼64	2659	13.66	4830	15.08		
	65∼	2492	12.80	3847	12.01		
Marital status	Unmarried	3597	18.48	7217	22.53	218.48	<0.001
	Married	14757	75.81	22376	69.85		
	Divorced	150	0.77	316	0.98		
	Bereft	962	4.94	2126	6.64		
Education	Primary school and below	6164	31.66	12121	37.84	1517.34	<0.001
	Junior school	5880	30.21	10086	31.48		
	Senior high/technicalsecondary school	3928	20.18	7441	23.23		
	Junior college	1631	8.38	1511	4.72		
	University and above	1863	9.57	876	2.73		
Occupation	Manager	625	3.21	574	1.79	10316.6	<0.001
	Business and service industry	3325	17.08	5044	15.75		
	Skilled labour	1718	8.82	1753	5.47		
	Clerk	985	5.06	1095	3.42		
	Worker	610	3.13	9736	30.39		
	Student	994	5.11	1746	5.45		
	Retired servant	2363	12.14	2061	6.43		
	Farmer	864	4.44	3625	11.32		
	Unemployed	4200	21.58	5741	17.92		
	Else	3782	19.43	660	2.06		
Family per capitamonthly income	Lower	8992	46.19	19739	61.62	1167.82	<0.001
	Higher	10474	53.81	12296	38.38		
Medical insurance	PEMI	103	0.53	1153	3.60	10894.2	<0.001
	MIE	1666	8.56	7817	24.40		
	MIURR	18469	94.88	15953	49.80		
	Self-pay	5742	29.50	18516	57.80		
	CMI	3543	18.20	1217	3.80		
	Others	1217	6.25	481	1.50		

Note: Following the international practice, 50% of the median or average income in one country or a particular area is always considered as the low income standard [39]; Medical insurance: PEMI: Public expense medical insurance; MIE: Medical insurance for employees; MIURR: Medical insurance for urban and rural residents; CMI: Commercial medical insurance.

### Prevalence of the five NCDs

The analysis showed that 2,786 of the rural residents aged ≥18 years had chronic diseases, with a prevalence of the five NCDs of 8.70% (2,786/32,035). A total of 2,443 urban residents aged ≥18 years had NCDs, with a prevalence of 12.55% (2,443/19,466). There was a significant difference between rural and urban areas in the prevalence of NCDs in general and for each NCD individually (*P*<0.001) [chi-square test] (Table 2).

**Table 2:** Prevalence of NCDs in rural and urban areas (n = number).

Area	Hypertension		Diabetes		COPD		Stroke		Heart disease		Total		?^2^	*P* value
	n	%	n	%	n	%	n	%	n	%	n	%		
Urban	1583	8.13	314	1.61	234	1.20	156	0.80	156	0.80	2443	12.55	132.98	<0.001
Rural	2084	6.50	300	0.94	84	0.26	110	0.34	208	0.65	2786	8.70		
?^2^	3667.0		614.0		318.0		266.0		364.0		5229.0			
*P* value	<0.001		<0.001		<0.001		<0.001		<0.001		<0.001			

### Selection of healthcare facilities

The results showed an interaction such that patients with the five NCDs in rural and urban areas selected different healthcare facilities for treatment. Most of the patients, in general, selected tertiary general hospitals or specialized hospitals or district-level or secondary hospitals for treatment. The proportion of participants who selected CHS facilities for treatment was considerably lower. Rural patients were more likely to select tertiary general hospitals or specialized hospitals than were urban residents (16.26% in rural and 9.78% in urban), and significance was found. There was a significant difference in the place at which NCDs were contracted (in urban area χ^2^ = 155.84 vs. in rural area χ^2^ = 281.19, *P<*0.001) and patients’ preference of healthcare facilities (in urban area χ^2^ = 23.39 vs. in rural area χ^2^ = 96.96, *P<*0.001) for CHS, district-level/secondary hospital and tertiary general/secondary hospital. For the place at which NCDs were contracted, there was a significant difference for hypertension and stroke between urban and rural areas (*P*<0.001) [chi-square test] (Table 3).

**Table 3:** Selection of healthcare facilities (n = number).

Items	Disease	Urban						Rural						?^2^	*P* value	
		CHS		DH/SH		TGH/SH		CHS		DH/SH		TGH/SH				
		n	%	n	%	n	%	n	%	n	%	n	%			
Healthcare facilities where NCDs were caught	Hypertension	415	26.21	749	47.32	419	26.47	673	32.29	957	45.92	454	21.79	19.87	<0.001	
	Diabetes	29	9.24	124	39.49	161	51.27	20	6.67	139	46.33	141	47.00	3.52	0.172	
	COPD	28	11.97	106	45.3	100	42.73	4	4.76	46	54.76	34	40.48	4.42	0.110	
	Stroke	29	18.59	62	39.74	65	41.67	2	1.82	46	41.82	62	56.36	18.56	<0.001	
	Heart disease	7	4.49	66	42.31	83	53.20	13	6.25	95	45.67	100	48.08	1.20	0.549	
	Total	508	20.79	1107	45.32	828	33.89	712	25.56	1283	46.05	791	28.39	25.53	<0.001	
	?^2^	155.84						281.19								
	*P* value	<0.001						<0.001								
Patients’ preference of healthcare facilities	Hypertension	647	40.87	773	48.83	163	10.3	1194	57.29	615	29.51	275	13.20	143.4	<0.001	
	Diabetes	107	34.08	184	58.60	23	7.32	118	39.33	110	36.67	72	24.00	44.14	<0.001	
	COPD	112	47.86	99	42.31	23	9.83	38	45.24	33	39.28	13	15.48	1.97	0.374	
	Stroke	77	49.36	65	41.67	14	8.97	38	34.55	33	30.00	39	35.45	28.36	<0.001	
	Heart disease	55	35.26	85	54.49	16	10.25	88	42.31	66	31.73	54	25.96	23.69	<0.001	
	Total	998	40.85	1206	49.37	239	9.78	1476	52.98	857	30.76	453	16.26	195.92	<0.001	
	?^2^	23.39						96.96								
	*P* value	0.009						<0.001								

Note: CHS: Community Healthcare service; DH/SH: district-level hospitals or secondary hospitals; TGH/SH: tertiary general hospitals or specialized hospitals.

In urban areas, patients preferred district-level or secondary hospitals over CHS at the first sign of illness or for minor health concerns, with the exception of COPD subjects. In rural areas, patients with all types of NCDs visited the CHS first and district-level hospitals or secondary hospitals later. These results indicate that the primary distinction between rural and urban patients is in their relative usage of the CHS. In terms of patients’ preference for healthcare facilities, there was a significant difference for hypertension, diabetes, stroke, and heart disease patients between urban and rural areas (*P*<0.001) [chi-square test] (Table 3).

### Rationale for selecting CHS facilities

Table 4 shows that patients with the five NCDs in the rural and urban areas visited CHS facilities for different reasons. In urban areas, patients with NCDs visited CHS facilities because these facilities were designated to provide healthcare under medical insurance coverage. Urban patients also considered the attitude and skill of medical staff, and the distance to CHS facility. They cared less about medical cost, waiting time, and familiarity with medical staff. However, in rural areas, the first factor considered by patients was distance, followed by the attitude of medical staff and medical skills. Rural patients did not consider whether services at the facilities were under medical insurance coverage. There was a significant difference between rural and urban areas on the rationale for selecting CHS facilities and the perception of problems in CHS facilities (*P<*0.001) [chi-square test] (Table 4).

**Table 4:** Rationale for selecting CHS facilities and Perception of problems in CHS facilities (n = number).

Items	Total (n = 5229)		Urban (n = 2443)		Rural (n = 2786)		?^2^	*P* value
	n	%	n	%	n	%		
**Rationale for selecting CHS facilities**								
Short distance	3158	60.39	1157	47.37	2001	71.84	986.57	<0.001
Good attitude for service	2125	40.64	1205	49.34	920	33.01		
Appointed healthcare facilities	1638	31.33	1286	52.63	352	12.62		
Reliable medical skills	1614	30.87	884	36.18	730	26.21		
Convenient procedures	1587	30.35	884	36.18	703	25.24		
Short time for waiting	1033	19.76	627	25.66	406	14.56		
Low price	883	16.89	450	18.42	433	15.53		
Be familiar with doctors and nurses	572	10.94	193	7.89	379	13.59		
Comfortable consulting environment	467	8.93	305	12.50	162	5.83		
Others	54	1.03	0	0.00	54	1.94		
**Perception of problems in CHS facilities**								
Obsolete equipment	1659	31.73	820	33.55	839	30.10	144.55	<0.001
Lack of drug varieties	1613	30.85	964	39.47	649	23.30		
Doubtful for medical skills	873	16.70	386	15.79	487	17.48		
Uncomfortable consulting environment	555	10.61	257	10.53	298	10.68		
Inconvenient procedures	172	3.29	64	2.63	108	3.88		
Others	151	2.89	97	3.95	54	1.94		
Bad attitude for service	140	2.68	32	1.32	108	3.88		
High price	134	2.56	80	3.29	54	1.94		
Non appointed healthcare facilities	59	1.13	32	1.32	27	0.97		

### Perception of problems in CHS facilities

When we investigated the reasons why patients decided against CHS facilities in favor of district-level hospitals or secondary hospitals and tertiary general or specialized hospitals, we found that, as a whole, patients cited four major factors: lack of drug variety, obsolete equipment, doubt about the skills of medical staff, and an uncomfortable environment (Table 4).

### Patient factors influencing initial treatment in the community

The results of the multivariate unconditional logistic regression demonstrate that, in both rural and urban areas, education level affected whether patients sought initial treatment in the community. For urban areas, influencing factors were mainly awareness factors, such as education level, physical exercise, healthcare knowledge, and community healthcare report. In rural areas, influencing factors included awareness factors education level, income level and medical insurance (Table 5).

**Table 5:** Patient factors influencing initial treatment in the community of urban and rural areas.

Area	Factors	OR	95.0% C. I	*P* value
Urban	Education			
	primary school and below (reference)			
	Junior college	0.261	0.070–0.976	0.046
	Physical exercise (did not attend vs. participated)	1.274	1.125–1.443	0.000
	Healthcare knowledge (did not obtain vs. initiatively obtain)	1.266	1.038–1.543	0.020
	Community health care report (did not taken vs. taken)	0.595	0.470–0.751	0.000
Rural	Education			
	primary school and below (reference)			
	Junior school	0.424	0.209–0.860	0.017
	Senior high/technical secondary school	0.409	0.193–0.870	0.020
	Junior college	0.206	0.053–0.794	0.022
	University and above	0.101	0.021–0.480	0.004
	Income level (lower vs. higher)	0.742	0.559–0.985	0.039
	Medical insurance (did not have vs. already have)	0.714	0.519–0.983	0.039

## Discussion

With the rapid development of China’s economy over the past 30 years, lifestyles of the Chinese population have significantly changed. Consequently, the major NCDs have become increasingly common [22] [23]. Our study included five common NCDs, which contributed to more than 80% of all deaths [24], representing a significant threat to public health in China.

To improve health status of patients with NCDs and to promote healthcare equity, it is of substantial importance to address chronic medical conditions through primary care initially at the community level. According to a previous study, only about 5% of all patients require diagnosis and treatment by a specialist, whereas more than 90% of chronic diseases could potentially be well-treated by a qualified GP [25]. Our results provided interesting findings with regard to the healthcare utilisation. There were 40.85% and 9.78% patients in urban area reported a willingness to use CHS and tertiary/secondary hospitals, respectively, for initial contact of care; however, the percentages of patients with NCDs who in fact used these facilities were 20.79% and 33.89%, respectively. The distinction in rural area was much sharper than that of urban area. The differences between NCDs patients’ preference of healthcare facilities and their actually selections were also striking. These findings indicate that there is still much progress to be made towards a more rational use of medical health resources. This applies to both urban and rural residents. In the meantime, significant differences in medical resource distribution between urban and rural areas would further weaken healthcare equity and affect accessibility and effectiveness of healthcare service for patients with chronic conditions.

Education level is associated with the choice of healthcare facilities in both rural and urban residents. In addition, physical exercise, healthcare knowledge, and community health knowledge are factors contributing to the choice of healthcare facilities among urban residents. It implies that lifestyles and health awareness might be underlying determinants among those with chronic diseases [26] [27] [28]. Similar association is also observed among rural residents in the study, although the impact is, to some extent, lessened than that among urban residents. This conclusion can also be drawn from the different choices for initial consultation: the percentage of patients choosing tertiary general hospitals, specialized hospitals, district-level, or secondary hospitals was lower among rural residents, whereas the percentage of those choosing a CHS facility was larger than that in the urban. From this perspective, the results are consistent with those reported by other studies [29] [30] [31].

By the comparison between rural and urban areas, it is clearly shown in the study that the choice of medical facilities is related to socioeconomic factors such as social medical insurance and income level. Previous literatures have demonstrated that low income is linked to unmet needs and poor access to healthcare [32]. Meanwhile, there were significant gaps between rural and urban residents in medical insurance coverage from Medical Insurance for Urban and Rural Residents (MIURR) and self-pay, which partly reflected a wide gap in socioeconomic factors between urban and rural areas. The implementation of different scope of reimbursement in different level medical facilities could guild the residents to access to different level medical facilities for treatment according to their own economic situation and health need, which were much more obvious in rural areas. Medical insurance has become the dominant influencing factor for rural areas toward reducing the economic chasm in healthcare equality. The results suggested that medical insurance plays a substantial role in the choice of medical facilities and could be one of the most important measures towards narrowing the gap in the utilization of medical service between urban and rural areas. Thus, the present study findings embrace the idea of combining initial consultation in the community and medical insurance to ensure that GPs serve as “gatekeepers” and patients are reasonably referred in the health system [33] [34] [35].

Our study indicates that the main reasons why patients with the five NCDs did not visit the CHS facilities for initial treatment were as follows: 1) lack of drug variety, 2) obsolete equipment, 3) doubts about healthcare professional’s medical skills, and 4) an uncomfortable consulting environment in both urban and rural areas. These reasons reflect the need for CHS facilities on constructing themselves. Also the results showed that the first rationale for selecting CHS facilities of rural patients was “short distance.” Though it obviously indicates the facilitation of CHS in meeting the demand in remote and rural areas, it still highlights the significant gap of medical resources from urban areas. The above results meant patients did not attend primary care services because of lack of credibility and/or perception of good quality care, particularly in a growing emerging paradigm of patient centered care it makes sense to link the aspirational goal with the perceived deficiencies of the PHC centers in China. It calls for a rise in the investment from the government, such as providing more human resources, increasing drug variety, and replacing obsolete equipment, in order to increase the attractiveness and acceptance of CHS facilities as the initial contact of care. This is particularly salient in remote and rural areas. International studies have demonstrated the effectiveness of these policies [36] [37] [38].

In China, the government aims to build a CHS network that will allow residents to access primary care within a 15-minute walking distance and to provide more reasonable medical coverage. This study shows that, whether in rural or urban regions, patients understand that there is a CHS center or station in their community, and further, that it is within close proximity. However, the results also demonstrate that the proximity of CHS facilities is not the deciding factor of initial consultation choice. Initial consultation in a CHS facility is important in that it provides disease screening and diagnosis, continuous intervention of unhealthy lifestyle and bad habits, and long-term care and maintenance for chronic patients. It can be foreseen that with the extension of medical insurance and reasonable distribution of healthcare resources, initial consultation at community-based primary care facilities would become possible and feasible, given that the primary care-oriented healthcare reform is continuously ongoing. Such a system would further control escalating unnecessary medical expenditure, prevent chronic illnesses through screening, and provide immediate diagnosis and proper management of identified conditions.

### Ethical Approval

All research activities were conducted with integrity and in line with generally accepted ethical principles and approved by the Ethics Committees of Tongji Medical College of Huazhong University of Science and Technology, and of Guangzhou Medical University. All participants read a statement that explained the purpose of the survey and gave written informed content before participation in the study. We conducted the survey under the agreement of the medical staff of the CHS facilities. None of the personal information of these medical staff involved in the survey is available to people outside of the study team.

### Limitations

First, the study was conducted within one province in south China, therefore, caution is advised when applying the results to other regions of China. However, the data were collected from a community-based sample, which significantly increases its representativeness.

## Conclusions

This study concludes that it is of substantial importance to establish an equal health system that adequately covers both urban and rural areas through expanding the coverage of medical insurance and a GP-based CHS network. Healthcare policies should be made for both rural and urban areas as a function of differing demographics, awareness characteristics, and socioeconomic factors.
